# Down‐regulation of MTHFD2 inhibits NSCLC progression by suppressing cycle‐related genes

**DOI:** 10.1111/jcmm.14844

**Published:** 2019-11-28

**Authors:** Chang Yu, Lehe Yang, Mengsi Cai, Feng Zhou, Sisi Xiao, Yaozhe Li, Tingting Wan, Dezhi Cheng, Liangxing Wang, Chengguang Zhao, Xiaoying Huang

**Affiliations:** ^1^ Key Laboratory of Heart and Lung Division of Pulmonary Medicine The First Affiliated Hospital of Wenzhou Medical University Wenzhou China; ^2^ Interventional Therapy Department The First Affiliated Hospital of Wenzhou Medical University Wenzhou China; ^3^ Department of Thoracic Cardiovascular The First Affiliated Hospital of Wenzhou Medical University Wenzhou China; ^4^ Chemical Biology Research Center School of Pharmaceutical Sciences Wenzhou Medical University Wenzhou China

**Keywords:** bioinformatics, cell cycle, methylenetetrahydrofolate dehydrogenase 2, non‐small cell lung cancer

## Abstract

Methylenetetrahydrofolate dehydrogenase 2 (MTHFD2) is a bifunctional enzyme located in the mitochondria. It has been reported to be overexpressed in several malignancies. However, the relationship between the expression of MTHFD2 and non‐small cell lung cancer (NSCLC) remains largely unknown. In this study, we found that MTHFD2 was significantly overexpressed in NSCLC tissues and cell lines. Knockdown of MTHFD2 resulted in reduced cell growth and tumorigenicity in vitro and in vivo. Besides, the mRNA and protein expression level of cell cycle genes, such as CCNA2, MCM7 and SKP2, was decreased in MTHFD2 knockdown H1299 cells. Our results indicate that the inhibitory effect of MTHFD2 knockdown on NSCLC may be mediated via suppressing cell cycle‐related genes. These findings delineate the role of MTHFD2 in the development of NSCLC and may have potential applications in the treatment of NSCLC.

## 1 INTRODUCTION

Lung cancer is a leading cause of cancer‐related death worldwide. More than 220 000 cases are expected to be newly diagnosed in the United States in recent years, and the 5‐year overall survival rate remains lower than most other types of cancer.^1^, ^2^ Non‐small cell lung cancer (NSCLC) is the most common histological type of lung cancer in the clinic. Although advances in the surgical techniques, systemic chemotherapy and immunotherapy have improved the clinical outcome for NSCLC patients, the prognosis still remains unsatisfactory.^3^, ^4^ Therefore, it is of great clinical value to elucidate the molecular mechanism underlying the progression of NSCLC, so as to identify more specific therapeutic targets and develop new modalities of treatment.

Mutation in the expression pattern of cancer‐related genes, which includes oncogenes and tumour suppressors, plays important roles in the tumorigenesis or tumour progression.^5^, ^6^ The microarray technology is a high‐throughput platform to analyse the gene expression profiling.^7^ Through using bioinformatics analysis, the microarray has been an effective strategy to obtain gene signature during tumorigenesis and identify molecular biomarkers for cancer patients.^8^ Therefore, to explore and identify new molecular signature of NSCLC using microarray‐based gene expression have a great appeal.

Mitochondrial methylenetetrahydrofolate dehydrogenase 2 (MTHFD2), a mitochondrial enzyme involved in the metabolism of folate,^9^, ^10^, ^11^ is a bifunctional enzyme located in the mitochondria with methylene dehydrogenase and cyclohydrolase activities.^12^, ^13^ More recently, studies showed that MTHFD2 could confer redox homeostasis and promote caners cell growth, metastasis and correlate with poor survival.^14^ MTHFD2 suppression decreased tumour burden and prolonged survival.^15^, ^16^ To date, the relationship between overexpression of MTHFD2 and the development of NSCLC is still unknown. In the current study, MTHFD2 was identified to be significantly up‐regulated in clinical NSCLC tissues based on the microarray. Functional assays with MTHFD2 silencing in NSCLC cell lines were performed to characterize the biological effects of MTHFD2 in NSCLC cell growth and tumorigenicity both in vitro and in vivo*.* The underlying mechanisms of MTHFD2 were then investigated by gene profiling with MTHFD2 knockdown in H1299 cells and further verified by qPCR and Western blot analysis.

## 2 MATERIALS AND METHODS

### 2.1 Patient tissues

With the approval of the Ethics Committee of the First Affiliated Hospital of Wenzhou Medical University and informed consent, human NSCLC tissues and their adjacent tissues were obtained from the First Affiliated Hospital of Wenzhou Medical University. Fresh tissues were immediately snap‐frozen and stored at −80°C, or fixed and embedded in paraffin.

### 2.2 Reagents and antibodies

Dulbecco's Modified Eagle Medium (DMEM) was purchased from Corning (#10‐013‐CVR). Roswell Park Memorial Institute (RPMI)‐1640 media were obtained from Thermo‐Fisher Scientific. Foetal bovine serum (FBS) was obtained from Ausbian (#VS500T). Streptomycin, penicillin and trypsin‐EDTA were obtained from GIBCO. Methylthiazolyldiphenyl‐tetrazolium bromide (MTT, #JT343) was purchased from Genview. Dimethyl sulfoxide (DMSO) was purchased from Sigma‐Aldrich Co. The Annexin V‐FITC Apoptosis Detection Kit (#88‐8007) was purchased from eBioscience. Primary antibodies used in the present study included: MTHFD2 (Sigma, #HPA049657), CCNA2 (Cell Signaling Technology, #4656), MCM7 (Cell Signaling Technology, #3735) and SKP2 (Abcam, ab183039). Second antibody horseradish peroxidase (HRP)‐conjugated donkey anti‐rabbit IgG and HRP‐conjugated goat anti‐mouse IgG were obtained from Proteintech.

### 2.3 Cell culture

The human NSCLC cell lines including A549, 95D, H460 and H1299 were purchased from ATCC. A549 and H1299 cells were maintained in high glucose DMEM with 10% FBS, 100 μg/mL streptomycin and 100 units/mL penicillin. 95D and H460 cells were cultured in RPMI‐1640 media with 10% FBS, 100 μg/mL streptomycin and 100 units/mL penicillin. Cells were cultured in a humidified atmosphere with 5% CO_2_ at 37°C.

### 2.4 Establishment of stable MTHFD2 knockdown cell lines

The short hairpin RNAs (shRNAs) targeting the mRNA sequence of MTHFD2 (shMTHFD2) and a negative control shRNA (shCtrl) were generated. The sequence of shMTHFD2 was AATGTGTTTGGATCAGTAT. A549 and H1299 cell lines were infected with the lentivirus knocking down MTHFD2 (LV‐shMTHFD2) or negative control (LV‐shCtrl). The lentivirus was packaged and purchased from GENECHEM using above corresponding sequences. Stably transfected cell lines were isolated based on the puromycin selection.

### 2.5 Cell proliferation assay

After being transfected, the cells were seeded into 96‐well plates for further incubation. Cells were counted daily using the Celigo Imaging Cytometer (Nexcelom Bioscience), and each experiment was performed in triplicates.

### 2.6 MTT assay

MTT assay was utilized to measure cell viability. Briefly, cells were seeded into 96‐well plates and cultured overnight. MTT solution (20 μL) was added to each well. After 4 hours additional incubation, 150 μL DMSO was added. Absorbance was measured at 490 nm with an Enzyme mark instrument (M2009PR, Tecan infinite).

### 2.7 Apoptosis assay

For apoptosis detection, cells (H1299‐LV‐shNC, H1299‐LV‐shMTHFD2; A549‐LV‐shNC, A549‐LV‐shMTHFD2) were seeded into 6‐well plates. After 2 days of incubation, cells were collected, washed twice in ice‐cold PBS and then stained with the Annexin V‐APC (eBioscience) according to the manufacturer's instructions. Data were analysed by flow cytometer (Millipore).

### 2.8 Colony formation assay

The bottom agar layer was added to each well by 0.5% agar and media solution until it is semi‐solid. And the top agar layer was made of 0.3% agar and media solution. Each cell line was seeded at 1000 cells/well on 6‐well plates at 37°C in 5% CO_2_ atmosphere overnight. The culture medium was replaced by the fresh medium every two days to keep cells growing for 2 weeks. After 2 weeks, the colonies were stained with GIEMSA and photographed.

### 2.9 Animal experiments

All animal experiments were performed in female BALB/c nude mice (4‐6 weeks) obtained from the Shanghai Slac Laboratory Animal Co. All mice were fed and treated according to the protocols approved by the Animal Care and Use Committees of Wenzhou Medical University. Cells at a density of 2 × 10^7^ cells/mL (H1299‐LV‐shCtrl and H1299‐LV‐shMTHFD2) were resuspended in 200 μL Matrigel and injected subcutaneously into the flanks of mice. 24 days after injection, tumour volume was measured with the vernier calipers every three or four days. The formula was V = π/6 × A×B × B, with A and B being the maximum and minimum diameter. Finally, mice were killed, and tumours were photographed, weighed and measured.

### 2.10 Tissue microarray and immunohistochemistry

A total of 150 clinical specimens consisted of lung cancer tissues and the surrounding adjacent tissues were collected after the surgery in the First Affiliated Hospital of Wenzhou Medical University. With the written informed consent of all participants, the research program was approved by the ethics committee of Wenzhou Medical University. Tissue microarray analysis was performed using the GeneChem Biotech. The expression of MTHFD2 was assessed by immunohistochemical staining using an anti‐MTHFD2 antibody (#HPA049657, Sigma) at a dilution of 1:50. The cytoplasmic staining results were evaluated based on the percentage of positive cells and the intensity of staining. The percentage of positive cells were given scores of 0 (no tumour cells stained), 1(1%‐25%), 2 (26%‐50%), 3 (51%‐75%) or 4 (76%‐100%). The intensity of staining was scored as 0 (no staining), 1 (light yellow), 2 (yellow) or 3 (brown). The score of intensity x proportion was divided into low expression (≤6) and high expression (>6).

### 2.11 RNA extraction and quantitative real‐time PCR

Total RNA was extracted by using the TRIzol reagent (Invitrogen). The cDNA of mRNA was synthesized using the M‐MLV Reverse Transcriptase (Promega). Real‐time PCR was performed with the Stratagene Mx3000P qPCR system (Stratagene‐Agilent) using SYBR Green mix (Takara). During the cDNA preparation, the mRNA level was normalized to GAPDH expression. Each sample was tested in triplicate. The primers used were summarized in Table S1.

### 2.12 RNA‐seq and bioinformatics analysis

Total RNA was extracted respectively from 15 pairs of lung cancer tissues and the surrounding normal tissues or H1299 cells by using Trizol reagent (Invitrogen). An Agilent 2100 instrument was used to control the quality of total RNA. Amplified RNA was obtained via a GeneChip 3’ IVT Express Kit. The amplified RNA was purified and fragmented, then hybridization at 45°C for 16 hours. The GeneChip was washed and stained with GeneChip Fluidics Station 450. GeneChip Scanner3000 was used to collect data. According to the manufacturer's instructions, the PrimeView Human Gene Expression Array (Affymetrix, #901838) was used to analyse the mRNA expression profiling. The data were summarized via the Expression Console™ software (Affymetrix), and further analysed using the GeneSpring GX (Affymetrix). Kyoto Encyclopedia of Genes and Genomes (KEGG) and Gene Ontology (GO) pathway analyses were processed by DAVID database (http://david.abcc.ncifcrf.gov/, database for Annotation, Visualization and Integrated Discovery). To build knowledge‐based networks, Ingenuity Pathway Analysis software was employed (IPA; Ingenuity Systems).

### 2.13 Western blot analysis

Total proteins were extracted from cells using ice‐cold Radio Immunoprecipitation Assay (RIPA) lysis buffer containing protease inhibitors. BCA Protein Assay Kit (Takara) was used to quantify protein concentration. Approximately 20 μg of total protein lysate was separated by 10% SDS‐polyacrylamide gel and transferred to nitrocellulose membrane. Membranes were blocked with 5% skimmed milk for 2 hours and then incubated overnight with primary antibodies. After three rinses, membranes were incubated with horseradish peroxidase‐conjugated secondary antibodies at room temperature for 1 hour. Signals were visualized with Enhanced Chemiluminescence Detection Kit (Pierce Biotechnology).

### 2.14 Statistical analysis

GraphPad Prism 7 (Graph Pad Software Inc) was used to analyse data. Data were expressed as mean ± SD of three independent experiments. Student's t test was used to compare two groups. *P*‐value < .05 was regarded as statistically significant.

## 3 RESULTS

### 3.1 Identification of MTHFD2 as a potential oncogene in human NSCLC

**Figure 1:**
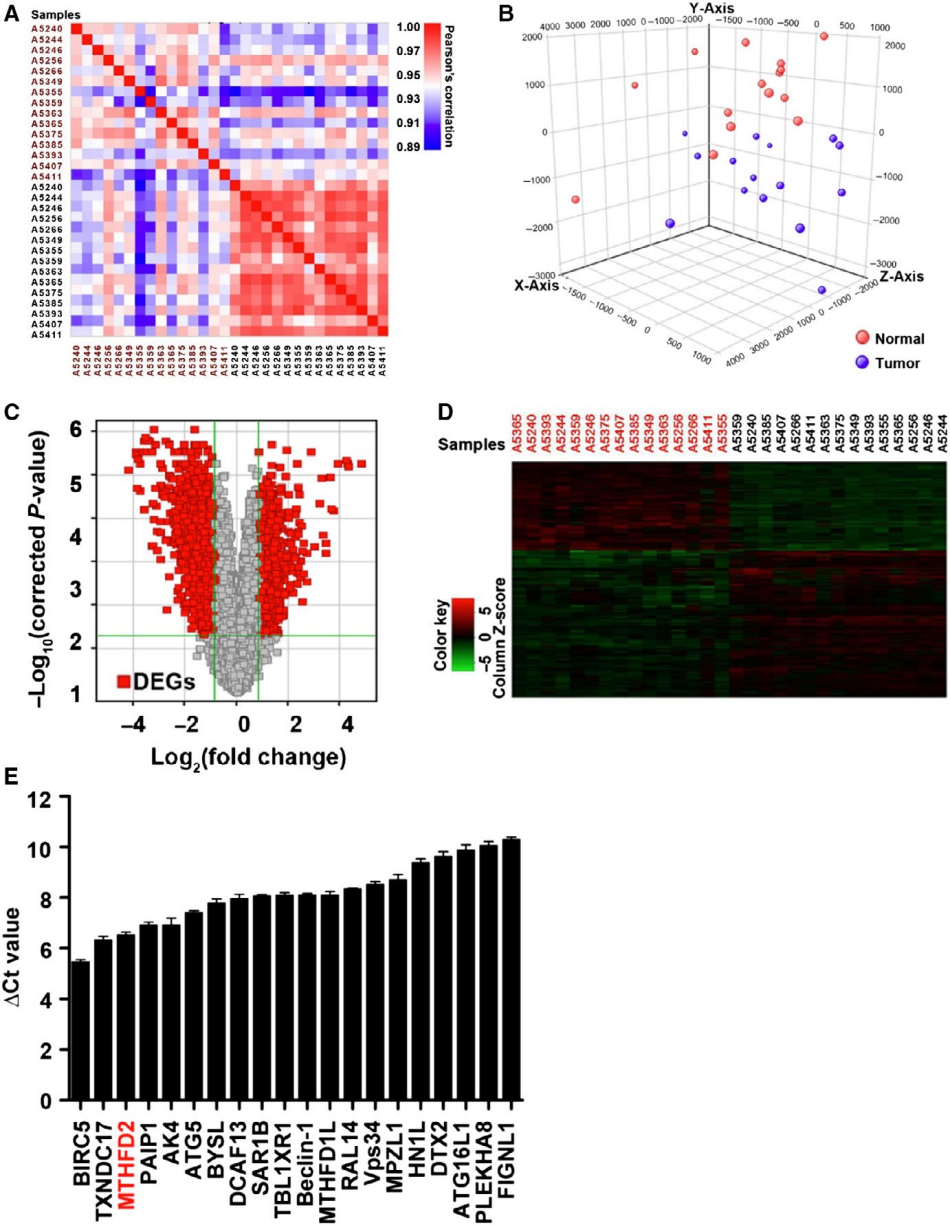
MTHFD2 is selected as a potential oncogene in human NSCLC. A, Pearson's correlation plot with hierarchical clustering of 15 pairs of NSCLC tissues (red) and corresponding adjacent non‐tumour tissues (black). B, Three‐dimensional principal component analysis for total transcriptional landscapes of 15 pairs of NSCLC tissues and corresponding adjacent non‐tumour tissues. C, Volcano plot of DEGs between NSCLC tissues and corresponding adjacent non‐tumour tissues. Red, significantly DEGs. Fold change > 1.8 and *P* < .05 were considered significant. D, Supervised clustering of genes identified from NSCLC tissues and corresponding adjacent non‐tumour tissues. E, qRT‐PCR was used to detect the mRNA expression of 20 candidates in H1299 cells. The mRNA level of each sample is normalized to that of GAPDH by the 2‐ΔCt method before comparative analysis

To identify dysregulated genes in NSCLC, the Human Gene Expression Array was employed to compare differentially expressed genes (DEGs) among 15 pairs of clinical NSCLC tissues and the corresponding adjacent non‐tumour tissues (Table S2). As shown in Figure 1A, global transcriptional states of theses tumour samples were distinct from the corresponding normal tissues that were highly consistent in distribution within normal groups. Additionally, the microarray similarity in each group is greater than that between tumour and normal samples (Figure 1B). Through using fold change > 1.8 and *P* < .05 as the threshold cut‐offs, in total, 1588 genes including 600 up‐regulated genes and 988 down‐regulated genes showed statistically significant differential expression in NSCLC samples (Figure 1C). As shown in Figure 1D, the supervised clustering of these DEGs identified was also exhibited. According to the literature search and functional prediction of these significantly up‐regulated DEGs, 20 highly abundant candidates in clinical NSCLC samples were chosen for further screening in H1299 cells using qRT‐PCR, and up‐regulated MTHFD2 which is a mitochondrial enzyme was ultimately selected for subsequent investigations (Figure 1E).

### 3.2 MTHFD2 is up‐regulated in NSCLC tissues and cells

**Figure 2:**
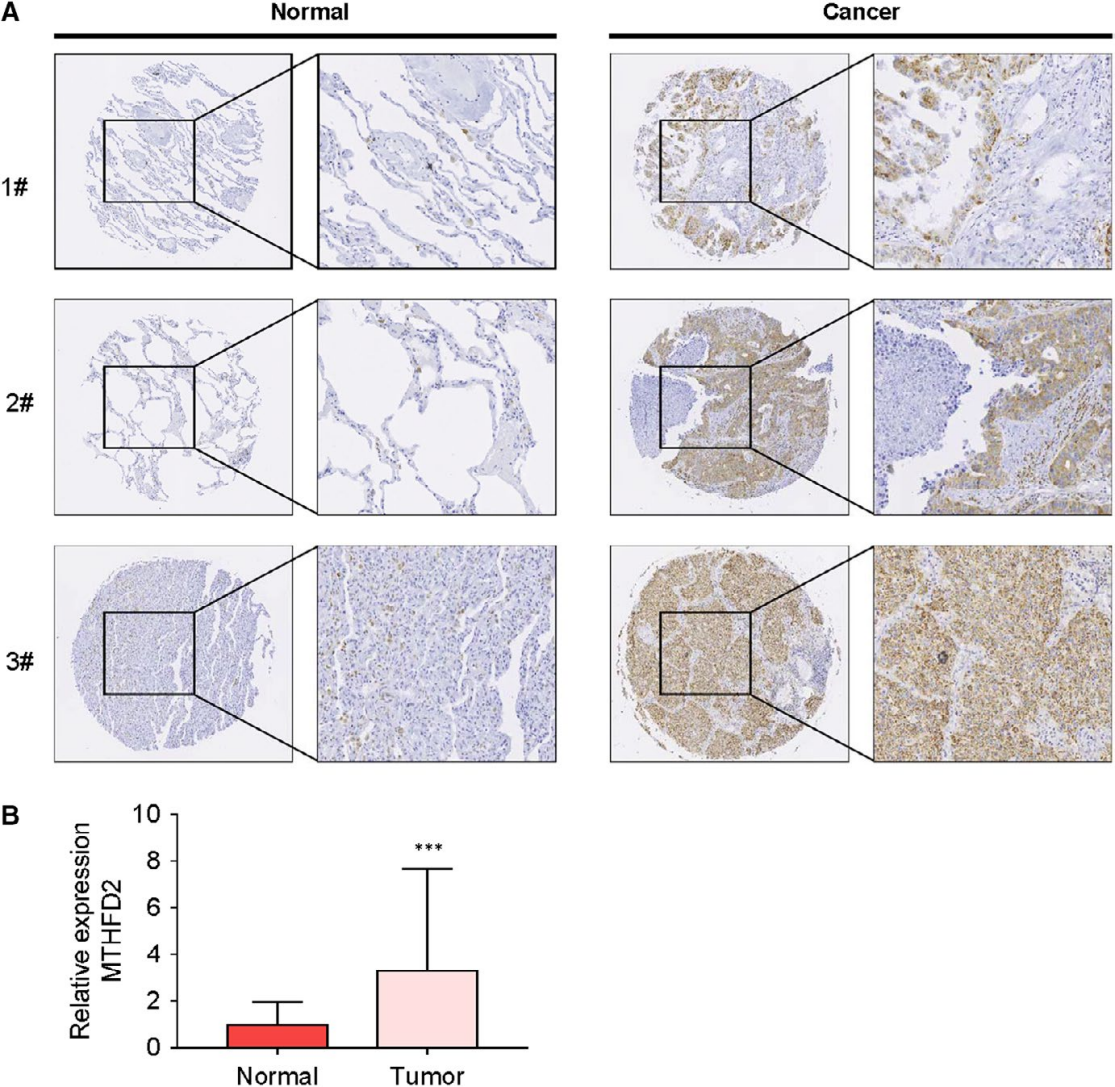
MTHFD2 is overexpressed in NSCLC tissue. A, Representative immunohistochemical staining of MTHFD2 on a tissue array containing100 clinical NSCLC tissues and 50 non‐tumour tissues. The cytoplasmic staining results were evaluated based on the percentage of positive cells and the intensity of staining. B, Quantification of MTHFD2 protein expression. The values are expressed as mean ± SD (n = 50 for non‐cancerous group and n = 100 for NSCLC group). *Significantly different from normal tissues, ***P < .001

**Figure 3:**
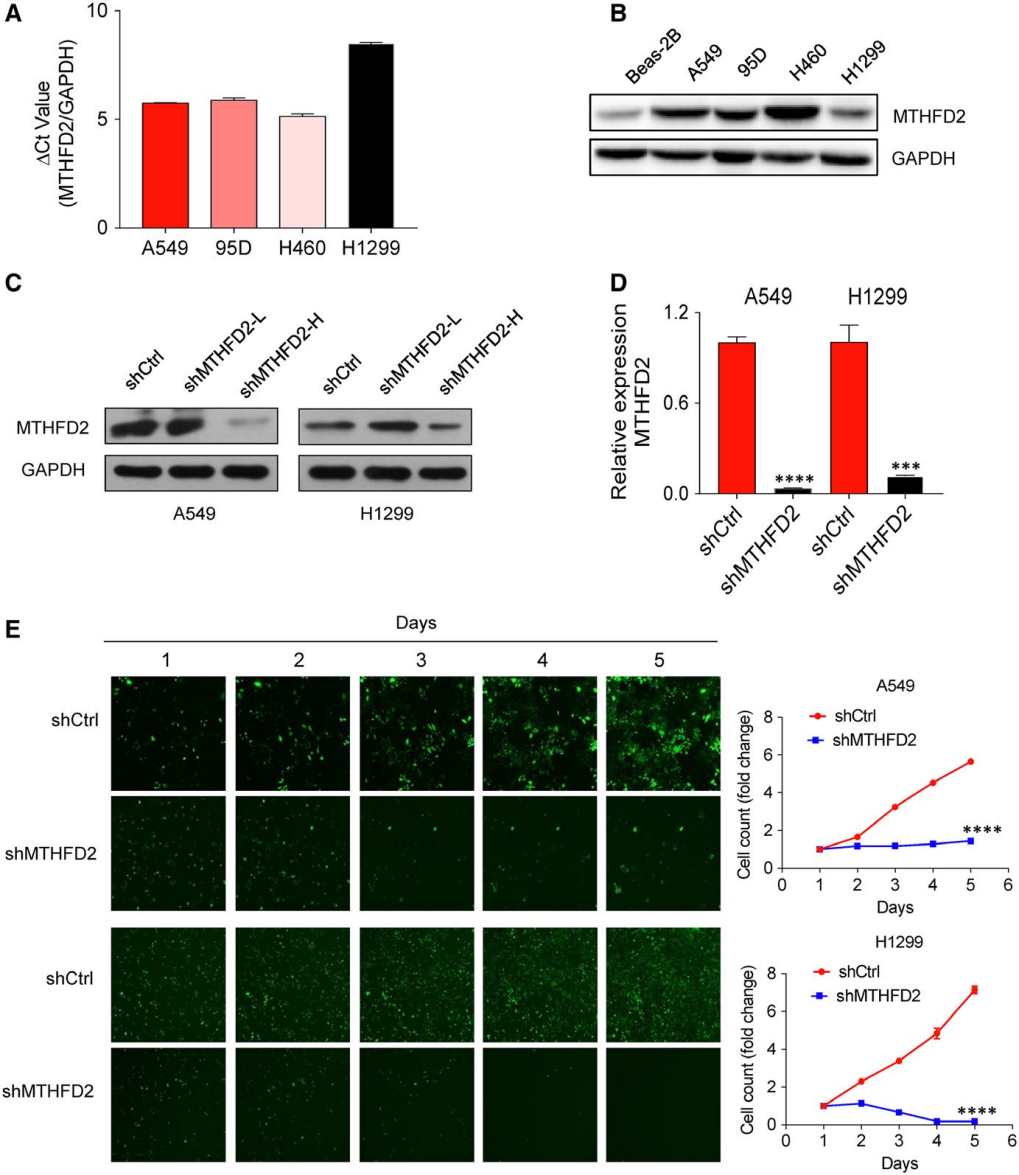
Knockdown of MTHFD2 inhibits NSCLC cell growth in vitro. A, qRT‐PCR was used to detect the mRNA expression of MTHFD2 in four NSCLC cell lines. The mRNA level of each sample is normalized to that of GAPDH by the 2‐ΔCt method before comparative analysis. B, MTHFD2 protein level was significantly overexpressed in NSCLC cells compared with that in lung normal epithelial cell Beas‐2B. C, Western blot analysis of MTHFD2 expression in MTHFD2‐silenced cells. Cells were respectively transfected with shMTHFD2 at a low MOI (shMTHFD2‐L) and high MOI (shMTHFD2‐H) for 48 h. GAPDH was used as a loading control. D, qRT‐PCR was used to detect the mRNA expression of MTHFD2 after transfection with shMTHFD2 at a high MOI for 48 h. The mRNA level is normalized to GAPDH by the 2^−ΔCt^ method before comparative analysis. E, Celigo cell counting analysis of cells transfected with shMTHFD2. Cell growth was measured using fluorescent photomicrographs every day for 5 d to capture the cells with green fluorescence. Growth curve was plotted by algorithms of the raw data of images (shCtrl vs shMTHFD2). The values are expressed as mean ± SD.*Significantly different from shCtrl, ***P < .001, ****P < .0001

To assess the roles of MTHFD2 in NSCLC, we first evaluated the protein expression of MTHFD2 in a cohort of 150 clinical samples consisted of 100 primary NSCLC tissues and 50 surrounding normal tissues (Table S3). As shown in Figure 2A,B, the protein level of MTHFD2 was significantly up‐regulated in NSCLC specimens when compared with normal lung tissues. Next, we measured the mRNA level of MTHFD2 by qRT‐PCR in NSCLC cells (Figure 3A). The mRNA level of each sample was normalized to that of GAPDH prior to comparative analysis using the 2‐ΔCt method. The ΔCt is equal to the difference between the ΔCt of MTHFD2 and GAPDH. The absolute value of ΔCt less than 12 is regarded to be expressive of high abundance, collectively, our data suggest that MTHFD2 is significantly up‐regulated in NSCLC. We also examined MTHFD2 protein expression in lung cancer cells and lung normal epithelial cells. The result showed that MTHFD2 protein level was significantly overexpressed in lung cancer cells compared with that in lung normal epithelial cell Beas‐2B (Figure 3B).

### 3.3 Knockdown of MTHFD2 inhibits the cell growth of NSCLC in vitro

**Figure 4:**
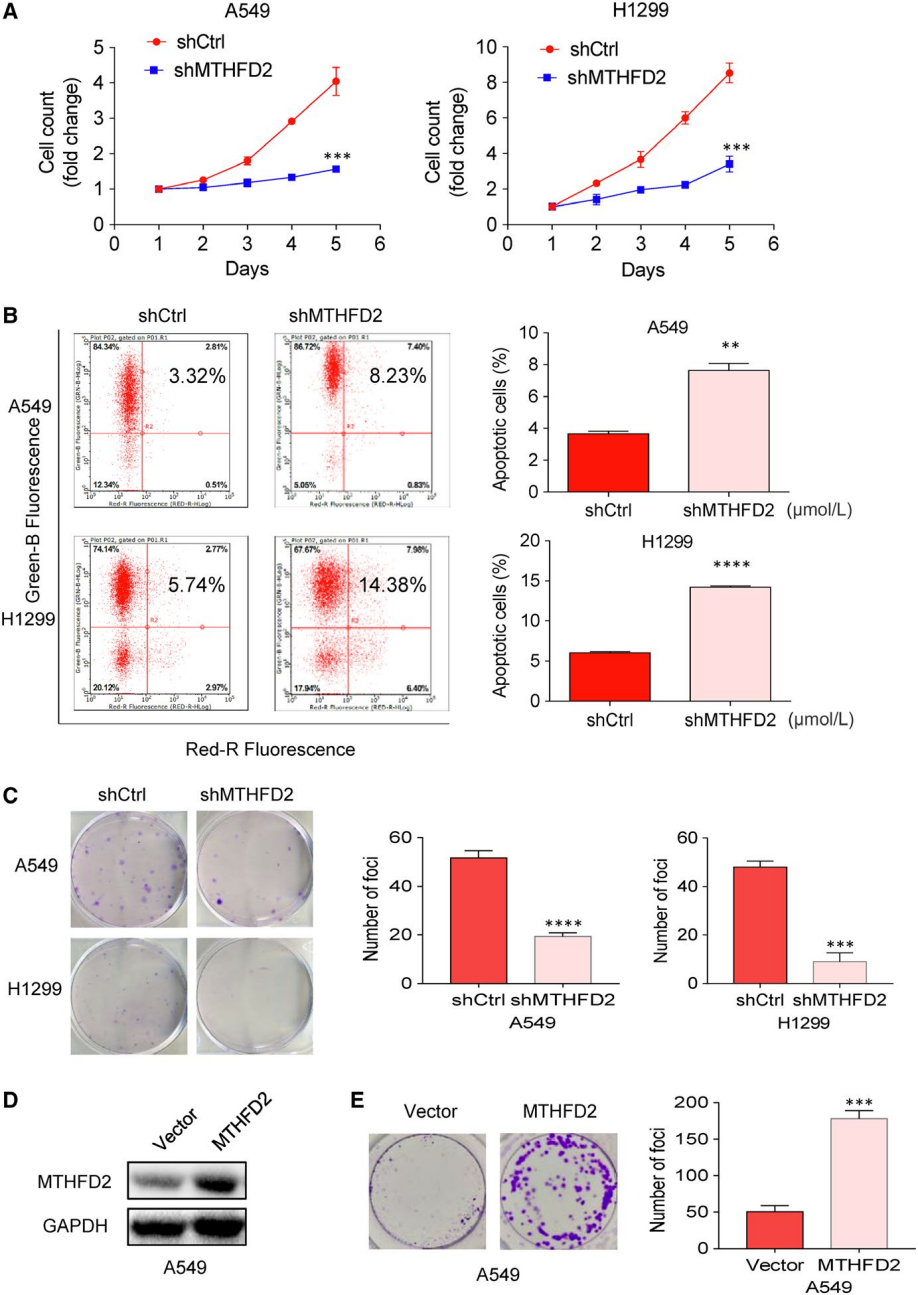
Knockdown of MTHFD2 inhibits proliferation and promotes apoptosis in NSCLC cells. A, Cell proliferation was determined by MTT assay in cells transfected with shMTHFD2 and shCtrl for 5 d. B, Cell apoptosis was measured using Annexin V staining and flow cytometry analysis in two groups of cells stably silencing MTHFD2. The horizontal coordinate was the signal value of Annexin V‐APC, and the vertical coordinate was the green fluorescence of the target gene virus infected in cells. C, Representative images of decreased colonies formation in monolayer culture induced by MTHFD2 silenced in NSCLC cells. The values are expressed as mean ± SD.*Significantly different from shCtrl, **P < .01, ***P < .001, ****P < .0001. D, Western blot analysis of MTHFD2 protein expression in A549 cells following MTHFD2 overexpression. E, Representative results of colony formation assay in MTHFD2‐overexpressing A549 cells. The quantitative numbers of colonies are shown at the right panel. The experiment was performed in triplicate wells in three independent experiments. *Significantly different from vector, ***P < .001

To explore the functional role of MTHFD2 in NSCLC, we transfected LV‐MTHFD2 shRNA or a non‐target control shRNA into H1299 and A549 to generate cells with MTHFD2 stably repressed or control cells, respectively. Cells were transfected with multiplicity of infection (MOI) (shMTHFD2‐L) and high MOI (shMTHFD2‐H), and shMTHFD2‐H significantly suppressed the protein expression of MTHFD2 when compared with shMTHFD2‐L in both A549 and H1299 (Figure 3C). Moreover, we also determined the mRNA level of MTHFD2 after transfection with shMTHFD2‐H (shMTHFD2) using qRT‐PCR and found that the mRNA level of MTHFD2 was also significantly inhibited (Figure 3D). To determine the role of MTHFD2 on cell proliferation, cells were transfected with shCtrl or shMTHMD2 and cultured for 5 days. Interestingly, MTHFD2 silencing significantly reduced the number of cells when compared with shCtrl in Celigo Cell Counting assay (Figure 3E), which was further confirmed by the results of MTT assay (Figure 4A). There were many cells floating in culture supernatant after shMTHFD2 transfection, flow cytometry was thus performed to examine whether MTHFD2 silencing induces apoptosis. As shown in Figure 4B, the percentage of apoptotic cells was significantly increased after shMTHFD2 transfection in both two cell lines. Taken together, these results suggest that MTHFD2 silencing inhibits the cell growth of NSCLC in vitro.

### 3.4 Knockdown of MTHFD2 inhibits the tumorigenicity of NSCLC

**Figure 5:**
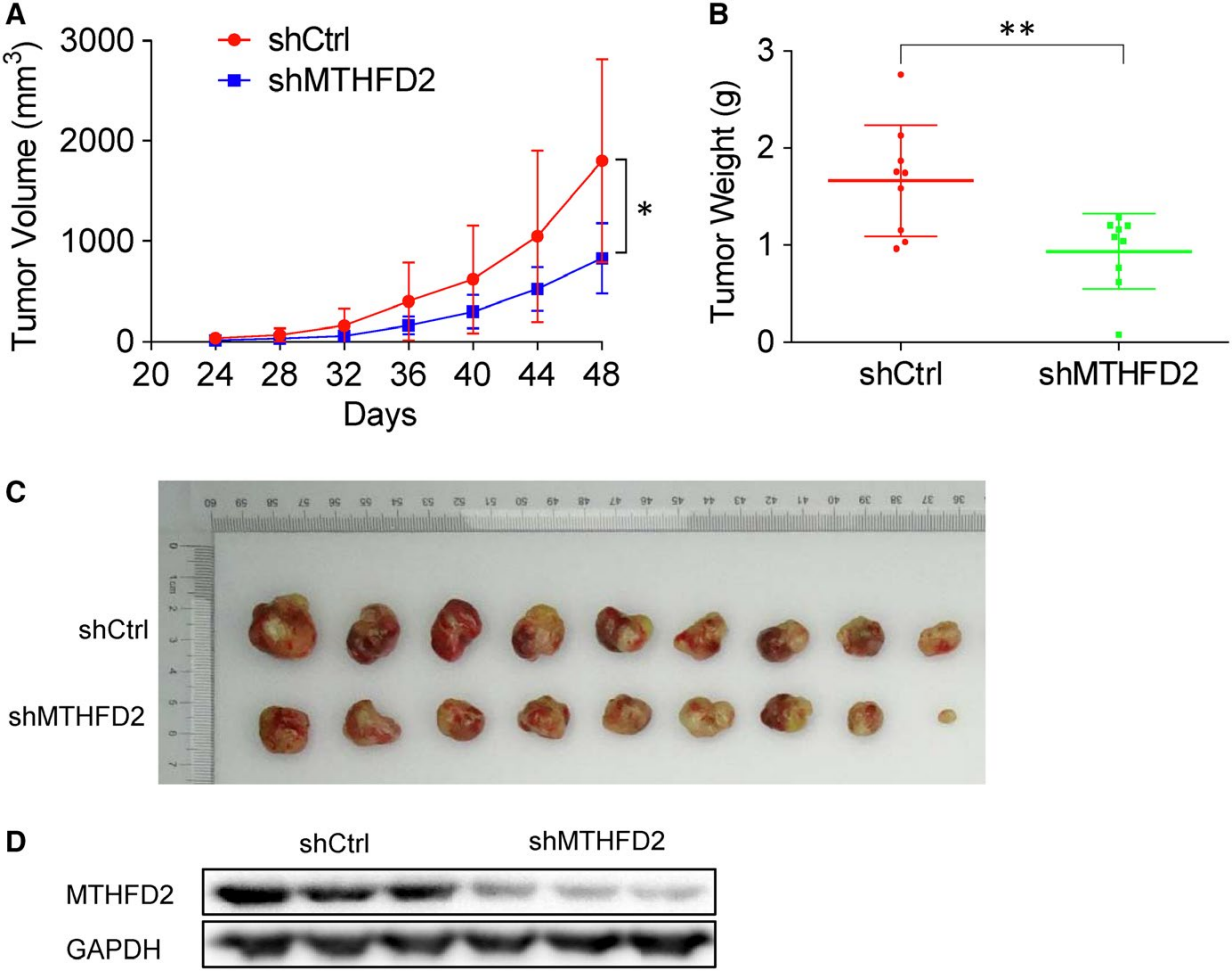
Knockdown of MTHFD2 inhibits the tumorigenicity of NSCLC. A, Images of the subcutaneous tumours formed in the nude mice after injection of shMTHFD2 and shCtrl transfected H1299 cells. B, Tumour growth curves revealed that xenograft tumour growth in nude mice was significantly slower in shMTHFD2‐treated group than that of shCtrl. C, Mean tumour weights 48 d after transplantation was shown. D, Western blot analysis of MTHFD2 in tumour xenograft tissues. The values are expressed as mean ± SD.*Significantly different from shCtrl, *P < .05, **P < .01

The ability to form colonies in an anchorage‐independent manner in soft agar cultures is one of the most significant characteristics of cancer cells. To evaluate the effect of MTHFD2 on colony formation of NSCLC, soft agar assay was performed with H1299 and A549 cells treated with MTHFD2 knockdown. Colony formation assay showed that MTHFD2 silencing significantly decreased the number of colonies when compared with control in H1299 and A549 cells (Figure 4C). In contrast, overexpression of MTHFD2 in A549 cells markedly enhanced the clonogenic ability compared with vector control cells (Figure 4D,E). To further evaluate the effect of MTHFD2 silencing on the tumorigenicity in vivo, H1299 cells transfected with shMTHFD2 were injected subcutaneously into nude mice. As shown in Figure 5, H1299 cells transfected with shMTHFD2 resulted in significant decrease in both tumour volume and weight in vivo as compared with control. Therefore, our data indicate that MTHFD2 plays an important role in colony formation and tumorigenicity of NSCLC cells.

### 3.5 mRNA profiling reveals down‐regulation of cell cycle‐related genes in H1299 cells with MTHFD2 knockdown

**Figure 6:**
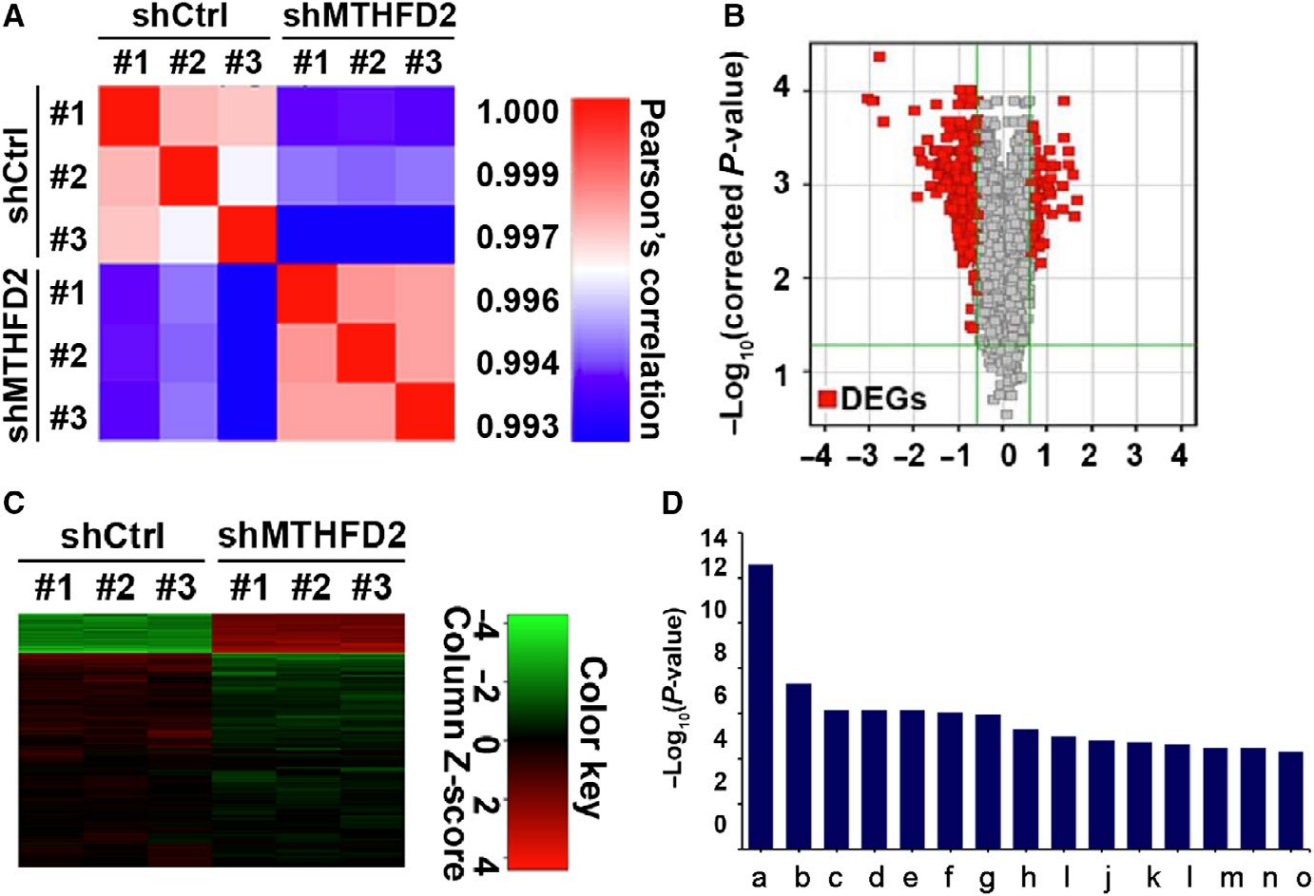
Deregulated genes in MTHFD2 knockdown H1299 cells. A, Pearson's correlation plot with hierarchical clustering of H1299 cells transfected with shMTHFD2 and shCtrl. B, Volcano plot of DEGs between shMTHFD2 group and shCtrl group. Red, significantly DEGs. Fold change > 1.5 and *P* < .05 were considered significant. C, Supervised clustering of genes identified from shMTHFD2 group and shCtrl group. D, Diseases and functional analysis of DEGs between shMTHFD2 and shCtrl by IPA software. a: cell cycle; b: DNA replication, recombination and repair; c: cancer; d: organismal injury and abnormalities; e: reproducitive system disease; f: neurological disease; g: cellular assembly and organization; h: organismal survival; i: gastrointestinal disease; j: cell death and survival; k: connective tissue disorder; l: cellular growth and proliferation; m: cellular development; n: endocrine disorders; o: reproductive system development and function

To explore the molecular mechanisms of MTHFD2 inhibiting cell proliferation and tumour growth, a genome‐wide mRNA screening was employed to compare gene expression profiles between MTHFD2 silencing group and control in H1299 cells. Global transcriptional state of cells with MTHFD2 silencing was largely distinct with shCtrl‐treated cells (Figure 6A). Through using the criteria of fold change > 1.5 and *P*‐value < .05, 501 DEGs were identified. Among these DEGs, 101 genes were up‐regulated, and 400 genes were down‐regulated (Figure 6B and Table S4), and the supervised clustering of these DEGs was also exhibited (Figure 6C). Furthermore, the diseases and functional analysis showed that the terms of cellular growth, proliferation and cancer were largely suppressed (Figure 6D). Altogether, our results indicate that the inhibitory effect of MTHFD2 knockdown on NSCLC may be mediated via suppressing cell cycle‐related genes.

### 3.6 Ingenuity pathway analysis of DEGs from H1299 cells with MTHFD2 knockdown

**Figure 7:**
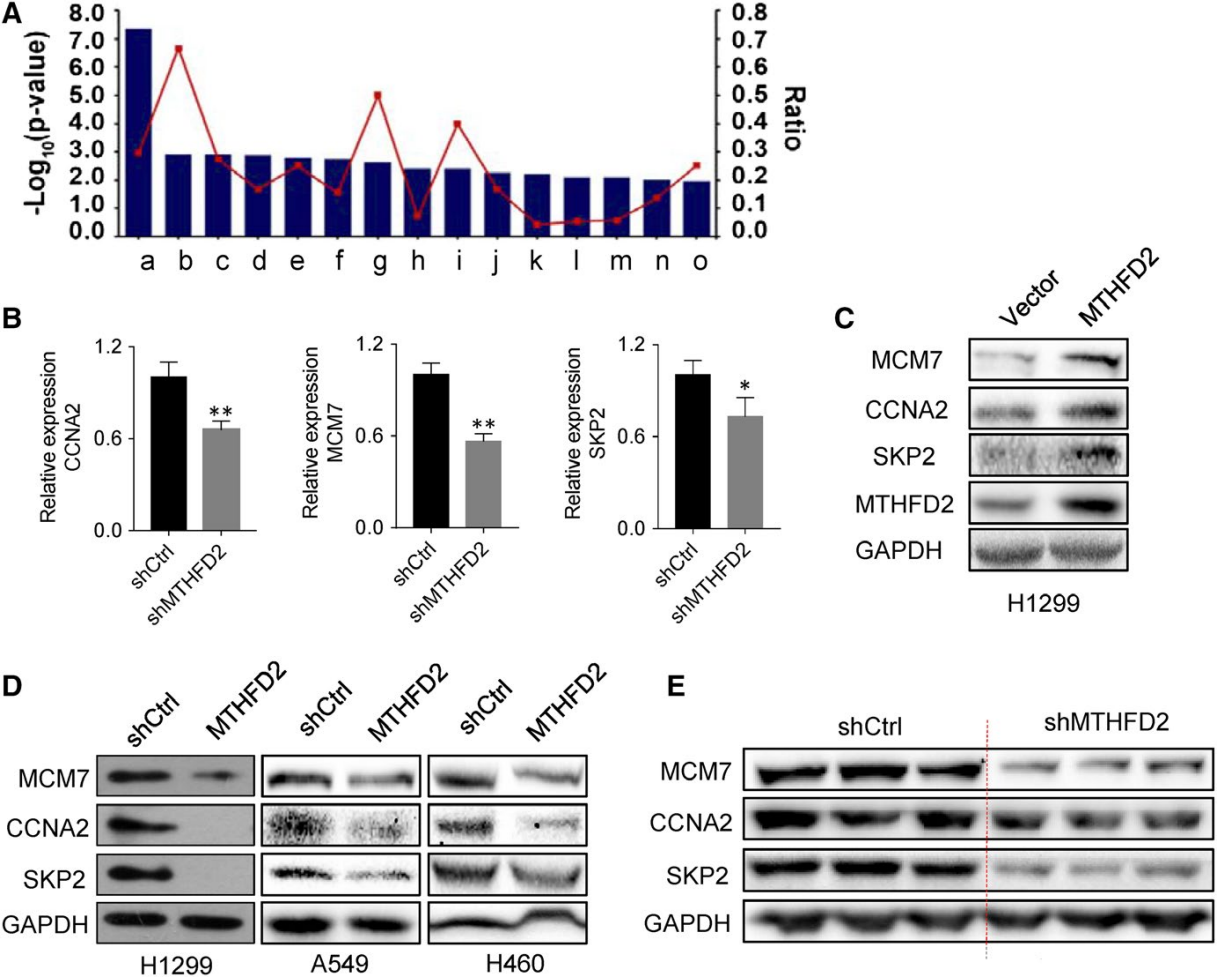
Identification of several genes as target of MTHFD2 in NSCLC. A, IPA canonical pathway analysis of DEGs between shMTHFD2 and shCtrl. On the horizontal axis, most significantly overrepresented pathways identified are exhibited, whereas the vertical axis shows the −Log_10_ of the p‐value calculated based on the Fisher exact test. The ratio reported as orange points represent the numbers of genes in a given pathway that meet cut‐off criteria divided by the total numbers of genes that make up that pathway. a: cell cycle control of chromosomal replication; b: 5‐aminoimidazole ribonucleotide biosynthesis I; c: purine nucleotides de novo biosynthesis II; d: oestrogen‐mediated S‐phase entry; e: cleavage and polyadenylation of pre‐mRNA; f: antiproliferation role of TOB in T‐cell signalling; g: Myo‐inositol biosynthesis; h: p53 signalling; i: tetrahydrofolate salvage from 5,10‐methenyltetrahydrofolate; j: D‐myo‐inositol(1,4,5)‐trisphosphate degradation; k: protein kinase A signalling; l: Wnt/β‐catenin signalling; m: aryl hydrocarbon receptor signalling; n: pyrimidine deoxyribonucleotides de novo biosynthesis; o: histidine degradation III. B, qRT‐PCR was used to detect the mRNA expression of 3 selected genes after transfection with shMTHFD2 and shCtrl in H1299 cells. The values are expressed as mean ± SD. *Significantly different from shCtrl, *P < .05, **P < .01. C, Western blot analysis of MTHFD2, CCNA2, MCM7 and SKP2 in MTHFD2‐overexpressing H1299 cells. D, Western blot analysis of CCNA2, MCM7 and SKP2 after transfection with shMTHFD2 and shCtrl in H1299 cells. E, Western blot analysis of CCNA2, MCM7 and SKP2 in tumour xenograft tissues

To further explore the regulatory pathways affected by these DEGs, we next performed the IPA functional analysis. Herein, canonical pathway analysis identified the S‐phase entry pathway (*Z *score < 2) was the most significantly suppressed signalling (Figure 7A).^17^ qRT‐PCR was used to verify the down‐regulation of 3 cell cycle‐related genes in H1299 cells after silencing MTHFD2 (Figure 7B). Besides, MTHFD2 overexpression increased the protein levels of CCNA2, MCM7 and SKP2 (Figure 7C). Conversely, the protein expression of CCNA2, MCM7 and SKP2 was significantly suppressed after MTHFD2 silencing (Figure 7D). Further, we assessed the protein levels of cycle‐related genes in tumour xenograft tissues. In accordance with our above results, Western blot analysis revealed that CCNA2, MCM7 and SKP2 expression were decreased in the MTHFD2 knockdown tumour tissues compared with shCtrl tumour tissues (Figure 7E). Altogether, these results suggest that MTHFD2 knockdown may inhibit cell proliferation and tumour growth via regulating cell cycle‐related genes.

## 4 DISCUSSION

Non‐small cell lung cancer is a widespread malignancy with increasing incidence rate which demands intensive investigation.^4^ Herein, we initially identify MTHFD2 is significantly up‐regulated in NSCLC as a potential oncogene. We also demonstrate that MTHFD2 plays an essential role in the development of NSCLC.

Methylenetetrahydrofolate dehydrogenase 2 was found to be co‐expressed with cell cycle proteins to progress cancer cell proliferation.^13^, ^18^ To explore the molecular mechanisms underlying the oncogenic role of MTHFD2, mRNA profiling was employed to obtain potential genes and pathways regulated by MTHFD2 via comparing DEGs between MTHFD silencing and empty virus‐transfected H1299 cells. By functional analysis, we identified cellular growth and proliferation to be highly suppressed, which may lead to the inhibitory effects of MTHFD2 silencing on NSCLC. Besides, we found that MTHFD2 knockdown significantly down‐regulated the mRNA and protein expression of cell cycle genes such as CCNA2, MCM7 and SKP2. Thus, our study found the cooperation between MTHFD2 and cell cycle genes in NSCLC development.^19^

Methylenetetrahydrofolate dehydrogenase 2 is a mitochondrial methylenetetrahydrofolate dehydrogenase and cyclohydrolase involved in one‐carbon metabolism. MTHFD2 plays a critical role in controlling N6‐methyladenosine (m6A) methylation of HIF‐2α levels and the oxidation of methylene‐THF to 10‐formyl‐THF in mitochondria, which results in promoted metabolic reprograming and tumour growth.^20^ In addition, MTHFD2‐dependent glycine synthesis is a prerequisite for angiogenesis.^10^ The exact mechanistic role of MTHFD2 in cancer is still a topic in the future. Given such important roles in the cancer cells proliferation, MTHFD2 has been recently considered to be a promising target for multiple types of cancer.^21^, ^22^, ^23^ During the treatments of acute myeloid leukaemia and colorectal cancer, targeting MTHFD2 can markedly suppress the tumour progression both in vitro and in vivo.^16^, ^21^ Importantly, our findings here extend the therapeutic function of MTHFD2 to NSCLC, which targeting MTHFD2 can be a potentially valuable approach in the clinic. Our findings also suggest that this enzyme may represent a novel therapeutic target for NSCLC treatment.

This finding provides additional evidence that MTHFD2 contributes to malignancy in NSCLC. It is possible that MTHFD2 plays important roles for conferring drug resistance in NSCLC.^6^ Moreover, MTHFD2‐mediated lung cancer cells resistance to gefitinib.^24^ KRAS mutation status is associated with the expression of MTHFD2 in lung cancer.^25^ MTHFD2 was also identified as a miR‐9 target gene that affects cell proliferation.^26^ Previously published studies have relied on shRNA or small molecule inhibitors directed suppression of MTHFD2.^14^, ^15^, ^16^, ^21^, ^24^ In future studies, it may also be informative to development of selective MTHFD2 inhibitors testing their effects in preclinical trials and the combinatorial effects with clinical chemotherapy drugs.

In summary, our preliminary study demonstrates that MTHFD2 is up‐regulated in NSCLC and plays important roles in the cell growth of NSCLC via promoting cell cycle genes expression. Our study provides a basis for utilizing MTHFD2 as a new diagnostic and therapeutic target in NSCLC.

## CONFLICT OF INTEREST

The authors declare no conflict of interest.

## AUTHOR CONTRIBUTIONS

C Yu and L Yang participated in the research design, performed most experiments, statistical analysis and paper writing; M Cai, F Zhou and S Xiao participated in the animal studies and Western blot analysis; Y Li and T Wan participated in the cell culture and immunofluorescence. D Cheng collected the human tissue samples. L Wang, C Zhao and X Huang designed and supervised the study and revised the manuscript. All authors read and approved the final manuscript.

## Data Availability

The data sets used and/or analysed during the current study are available from the corresponding author on reasonable request.
