# Supplementary material for: Down‐regulation of MTHFD2 inhibits NSCLC progression by suppressing cycle‐related genes
Source: J Cell Mol Med. 2019 Nov 28;24(2):1568–77. doi: 10.1111/jcmm.14844 (PMC6991687; doi:10.1111/jcmm.14844)
Supplement: Supplementary file 1 [file JCMM-24-1568-s001.docx]

**Table S1: Primers for real-time PCR analysis**

| **Gene** | **Forward** | **Reverse** |
| --- | --- | --- |
| GAPDH  MTHFD2  DBF4  MCM4  SKP2  CCNA2  E2F8  RRM2  MAD2L1  MCM7  CCND1  MCM10  THBS1  MCM2 | TGACTTCAACAGCGACACCCA  GATCCTGGTTGGCGAGAATCC  ATTTGCACAAACCTTGGGTC  TATCTAAGGGCAAAACAC  TGTTTGTAAGAGGTGGTATCG  CCCAGAAGTAGCAGAGTTTGTG  AAGTACGCCGAGCAGATTATG  AAGAAACGAGGACTGATGC  GAGTCGGGACCACAGTTTAT  ACATCACAGCAGCATACG  GGTGGCAAGAGTGTGGAG  GAAGAAGGTTACGCCACAGAG  AGACTCCGCATCGCAAAGG  ATGATCGAGAGCATCGAGAACC | CACCCTGTTGCTGTAGCCAAA  TCTGGAAGAGGCAACTGAACA  CTGGGATGAGGTGAAGTGGT  CAGTCACTGTCAGGAAAT  ACAGTATGCCGTGGAGGG  TTGTCCCGTGACTGTGTAGAG  ATGTCTGGGTGTCCATTTGGG  CTGTCTGCCACAAACTCAA  TTTTGTAGGCCACCATGCTA  TCCACCACATCCACCATT  CCTGGAAGTCAACGGTAGC  TTTACAGGTTCCCAGGTCAAG  TCACCACGTTGTTGTCAAGGG  GCCAAGTCCTCATAGTTCACCA |
