# Supplementary material for: Down‐regulation of MTHFD2 inhibits NSCLC progression by suppressing cycle‐related genes
Source: J Cell Mol Med. 2019 Nov 28;24(2):1568–77. doi: 10.1111/jcmm.14844 (PMC6991687; doi:10.1111/jcmm.14844)
Supplement: Supplementary file 2 [file JCMM-24-1568-s002.docx]

**Table S2 The clinicopathologic characteristics of samples (15 pairs)**

| **Number** | **Age** | **Sex** | **TNM** | **Histological subtype** | **EGFR mutation** |
| --- | --- | --- | --- | --- | --- |
| 1 | 49 | Male | \| T1bN0M0 \| \| --- \| | Adenocarcinoma | Undetected |
| 2 | 66 | Male | T3N0M0 | Squamous cell carcinoma | No |
| 3 | 62 | Female | T2N0M1 | Adenocarcinoma | Yes |
| 4 | 72 | Female | T1cN1M0 | Adenocarcinoma | Yes |
| 5 | 75 | Male | T2aN2M0 | Adenocarcinoma | No |
| 6 | 67 | Female | T1bN0M0 | Adenocarcinoma | No |
| 7 | 74 | Female | T4N1M1 | Squamous cell carcinoma | No |
| 8 | 66 | Female | T1bN0M0 | Adenocarcinoma | Yes |
| 9 | 69 | Female | T1cN0M0 | Adenocarcinoma | Yes |
| 10 | 68 | Male | T1bN0M0 | Adenocarcinoma | Undetected |
| 11 | 54 | Male | \| T1bN0M0 \| \| --- \| | Adenocarcinoma | Yes |
| 12 | 77 | Male | T1bN0M0 | Adenocarcinoma | No |
| 13 | 60 | Male | T1aN0M0 | Adenocarcinoma | Undetected |
| 14 | 54 | Male | \| T1bN1M0 \| \| --- \| | Adenocarcinoma | No |
| 15 | 58 | Female | T2N1M0 | Squamous cell carcinoma | No |
